# Comparative Effectiveness of Different Oral Antibiotics Regimens for Treatment of Urinary Tract Infection in Outpatients: An Analysis of National Representative Claims Database

**DOI:** 10.1097/MD.0000000000000304

**Published:** 2014-12-02

**Authors:** Meng-Tse Gabriel Lee, Shih-Hao Lee, Shy-Shin Chang, Si-Huei Lee, Matthew Lee, Cheng-Chung Fang, Shyr-Chyr Chen, Chien-Chang Lee

**Affiliations:** From the Department of Emergency Medicine, National Taiwan University Hospital, Taipei, Taiwan (M-TGL, Sh-HL, C-CF, S-CC, C-CL); Department of Family Medicine, Chang Gung Memorial Hospital, Taoyuan, Taiwan (S-SC); Graduate Institute of Clinical Medical Sciences, College of Medicine, Chang Gung University, Taoyuan, Taiwan (S-SC); Department of Rehabilitation and Physical Medicine, Taipei Veteran General Hospital (Si-HL); Department of Rehabilitation and Physical Medicine, National Yang-Ming University, Taipei, Taiwan (Si-HL); Medical Wisdom Inc, US (ML); and Department of Emergency Medicine and Department of General Medicine, National Taiwan University Hospital Yunlin Branch, Douliou, Taiwan (C-CL).

## Abstract

There are very limited data on the postmarketing outcome comparison of different guideline antibiotic regimens for patients with urinary tract infections (UTIs).

We carried out a population-based comparative effectiveness study from year 2000 through 2009, using the administrative data of 2 million patients from the National Health Informatics Project of Taiwan. Treatment failure was defined as either hospitalization or emergency department visits for UTI. Odd ratios were computed using conditional logistic regression models matched on propensity score.

We identified 73,675 individuals with UTI, of whom 54,796 (74.4%) received trimethoprim–sulfamethoxazole (TMP-SMX), 4184 (5.7%) received ciprofloxacin, 3142 (4.3%) received levofloxacin, 5984 (8.1%) received ofloxacin, and 5569 (7.6%) received norfloxacin. Compared with TMP-SMX, the composite treatment failure was significantly lowered for norfloxacin in propensity score (PS) matching analyses (OR, 0.73; 95% CI, 0.54–0.99). Both norfloxacin (PS-matched OR, 0.68; 95% CI, 0.47–0.98) and ofloxacin (PS-matched OR, 0.70; 95% CI, 0.49–0.99) had significantly lowered composite treatment failure rate when compared with ciprofloxacin. Subgroup analysis suggested that both norfloxacin and ofloxacin were more effective in female patients without complications (W/O indwelling catheters, W/O bedridden status and W/O spinal cord injury), when compared with either TMP-SMX or ciprofloxacin.

Among outpatients receiving oral fluoroquinolone therapy for UTIs, there was evidence of superiority of norfloxacin or ofloxacin over ciprofloxacin or TMP-SMX in terms of treatment failure. Given the observational nature of this study and regional difference in antibiotic resistance patterns, more studies are required to validate our results.

## INTRODUCTION

Urinary tract infections (UTIs) are a major cause of morbidity, and healthcare resource expenditure. In the United States, there are at least 8 million annual incidences of UTI, which results in an estimated cost of $2.14 billion.^1,2^ Women are more susceptible to UTIs and have a 50% chance of experiencing at least one episode of UTI during their lifetime.^2–5^ According to the Infectious Diseases Society of America (IDSA) consensus UTI treatment guidelines, Trimethoprim–sulfamethoxazole (TMP-SMX) and fluoroquinolones can both be used in different types of UTIs.^6,7^ Randomized controlled trials (RCT) have demonstrated that TMP-SMX and fluoroquinolones have equal efficacy in both complicated and uncomplicated UTIs.^8–14^ However, RCT cannot reflect the comparative effectiveness of these regimens in real-world settings.^15^ Most clinical trials have a small sample size and exclude elderly patients with multiple comorbidities.^16,17^

Unfortunately, as far as we are aware of, there is no comparative effectiveness study of different fluoroquinolone subgroups to TMP-SMX. Information on the comparative effectiveness between different fluoroquinolone subgroups is important for clinical decision. Both patients and insurance companies will like to find out whether a more advanced and more expensive fluoroquinolone subgroup can result in a better clinical outcome. Furthermore, the recent safety concerns for certain patients using respiratory fluoroquinolones, makes answering this comparative effectiveness question even more crucial.^18,19^ We proposed to perform an observational study in a population-based health claims database that reflects the real world of daily practice. Specifically, we assessed the effect of oral fluoroquinolone therapy compared with TMP-SMX on overall treatment failure in outpatients. Treatment failure results in persistence or progression of infection and increases risk of morbidity and mortality. Rates of treatment failure are operationalized as follow-up emergency department (ED) presentation, or hospitalization for UTI.

## MATERIALS AND METHODS

### Data Source

Taiwan has a government operated universal healthcare with coverage rate at 99.7% for it's 23 million residents. The database contains administrative and demographic information (sex, age, type of insurance plan, eligibility date, and income) as well as records of reimbursement for services rendered by physicians, in addition to diagnostic services (eg, laboratory, radiology), and records of prescription pharmaceutical usage (de-identified prescribing physician, quantity and date dispensed, and days’ supply) by residents of Taiwan. Several studies have already shown that this database is appropriate for use in pharmacoepidemiologic research.^20–22^ The institutional ethical committee of National Taiwan University Hospital has approved our study to look at a representative sample of 2,000,000 participants that was longitudinally followed from year 2000 to 2009.

### Identification of Cohort and Antibiotic Exposure

A new-user incident-case cohort design was adopted to identify UTI outpatients that were 18 years or older from January 2006 to November 2009. This design excluded any existing users of UTI-related antibiotic (TMP-SMX and ciprofloxacin, levofloxacin, ofloxacin, norfloxacin) and any prevalent cases of UTI 180 days before the index date (the first day of initial UTI diagnosis). International Classification of Disease 9th Clinical Modification (ICD-9-CM) code relevant to UTI used by published studies, are used to identify UTI patients.^23,24^ Specifically, 590.xx (infection of the kidney), 595.xx (cystitis) and 599.xx (other disorders of the urethra and urinary tract, including urinary tract infection of unspecified site) were used for case identification. Patients with unusual prescription length (>15 days) of antibiotics were also excluded. Finally subjects were followed up for 42 days postindex date, unless censored by death or termination of medical insurance.

### Identification of Treatment Failure

Treatment failure (or lack of effectiveness) was determined based on the presence or absence of further hospitalization/ emergency department visit consistent with the initially identified UTI infection. Specifically, hospitalization/ emergency department visit has to be more than 3 days but is within 42 days of index date.

### Identification of Covariates

We evaluated the following factors as potential confounders between treatment groups: age, sex, calendar year, geographic area, specialty of prescribing physicians, personal income, burden of comorbid conditions, potential risk factors for treatment failure, frequency of health care utilization 1 year before the index date, and use of specific medications 1 year before the index date. The covariates have been assessed for a total of 7 years from the patient index date. We used a combined weighted comorbidity index to control for each individual burden of comorbidity. This score combines the Charlson Index with Elixhauser system to offer improvements in comorbidity summarization. Specific comorbid conditions that may predispose to frequent UTI and precipitating factors potentially associated with treatment failure are collected and shown on Table 1. Information on use of specific medications was also collected 1 year before the index date (Table 1). Utilization of healthcare facilities may reflect the general health and severity of chronic disease that were not readily captured by ICD-9 codes. To correct for severity of chronic disease, we calculated the number of out patient department visit, number of emergency department visit, and number of hospitalization 1 year before the index date. In addition, information on the severity of UTI was captured by the procedure codes including intravenous infusion, injection, complete blood count, sedimentation rate testing, blood culture, urinalysis, urine catheterization, cystostomy, nephrostomy, renal sonography, CT, CRP measurement, and procalcitonin measurement.

**TABLE 1:**
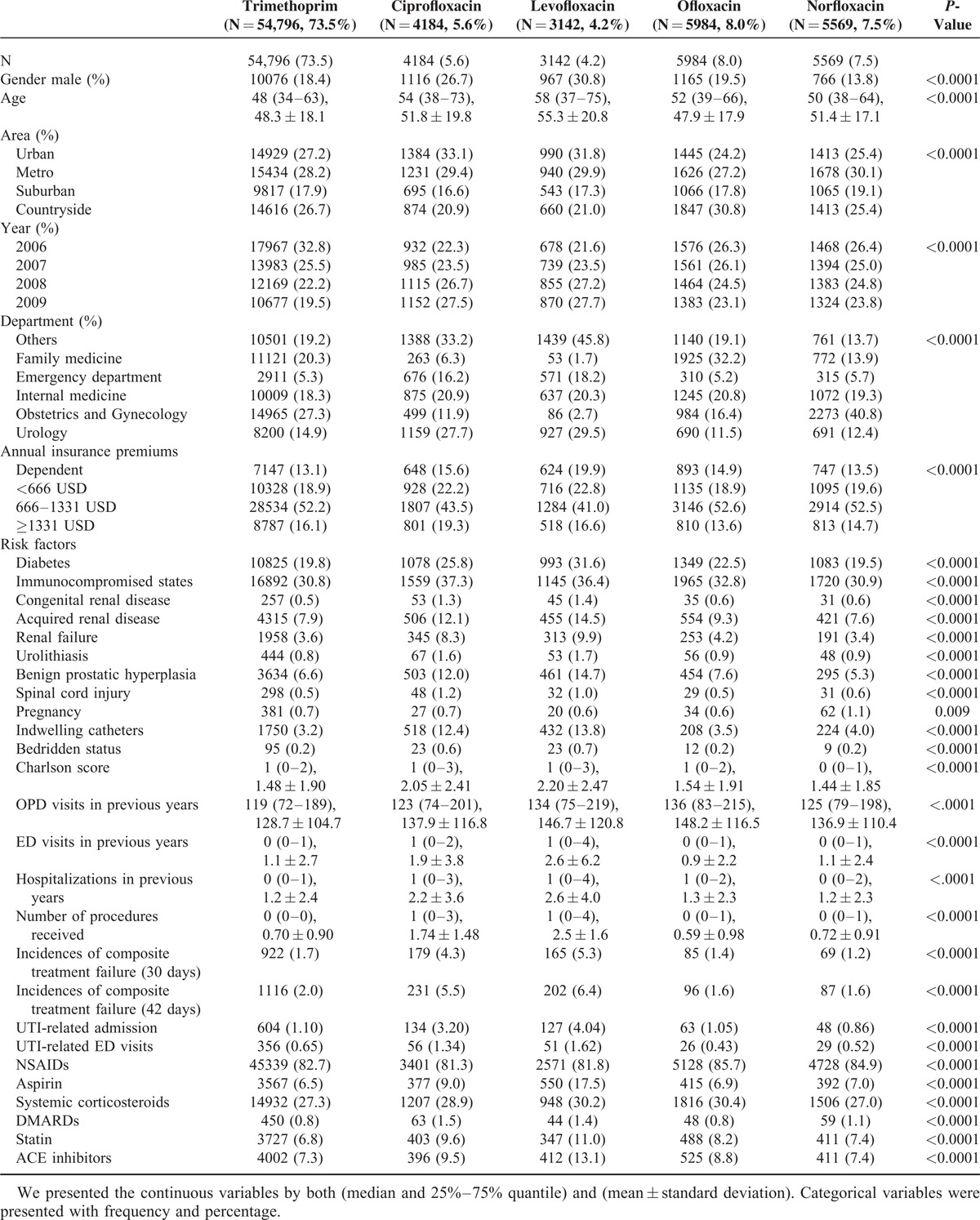
Characteristics of Patients With Urinary Tract Infection Receiving Different Antibiotic Regimens

### Statistical Analysis

Baseline subject characteristics were described and compared among users of different antibiotic regimens. We presented the continuous variables by both (median and 25%–75% quantile) and (mean ± standard deviation). Categorical variables were presented with frequency and percentage. Comparison of characteristics was assessed with Kruskal–Wallis tests for continuous variables, and Pearson chi-square tests for categorical variables. Multivariate binary logistic regression was conducted to examine the independent effect of different fluoroquinolones on the treatment failure outcome. The model was built by including the composite outcome of UTI treatment failure as dependent variable and all relevant covariates (Table 1) as the independent variables.

In addition, we estimated propensity scores (PS) for different antibiotic treatment using TMP/SMX or ciprofloxacin as the reference groups. The PS model (Appendix 1) was built with logistic regression model, with prescription of antibiotics as the dependent outcome and all covariates (Table 1) selected based on the literature and clinical judgment as the independent predictors. Dependent variables were antibiotic treatment received and the predictors were all the covariates mentioned above. We examined the distribution of PS in the study population and checked the balance of each covariate. PS matching of the patients is carried out using the greedy matching algorithm without any trimming. Outcomes between the 2 treatment groups were then compared in the patients after matching. To explore whether there are favorable antibiotics in different susceptible patient groups, we performed subgroup analysis. All P values are two-tailed. Statistical analysis was carried out using Statistical Analysis System software (SAS Institute, Version 9.2).

## RESULTS

### Participant Enrollment and Baseline Characteristics

The baseline characteristics of the participants receiving different UTI antibiotics are shown in Table 1. We identified 73,675 patients meeting inclusion criteria, of whom 54,796 (74.4%) received TMP-SMX and 18,869 (25.6%) received fluoroquinolone. Approximately 18.9% of study patients were male. Of the 18,869 patients receiving a fluoroquinolone, 4184 (5.7%) received ciprofloxacin, 3142 (4.3%) received levofloxacin, 5984 (8.1%) received ofloxacin, and 5569 (7.6%) received norfloxacin. TMP-SMX users are generally younger (48.3 ± 18.1 years old) than fluoroquinolone user (except for ofloxacin subgroup). There are differences in the average age of fluoroquinolone user, with ofloxacin having the youngest user (47.9 ± 17.9 years old) while levofloxacin (55.3 ± 20.8 years old) having the oldest users. We found that fluoroquinolones were generally more frequently prescribed in the urban area. From 2006 to 2009, there is a trend of decreasing prescription of TMP-SMX. The insurance premium categories do not seem to be associated with preferred use of certain kinds of antibiotics. Physicians of different specialties have different preference of antibiotic prescription. The family doctors tend to prescribe more ofloxacin, emergency department doctors prescribed more ciprofloxacin and levofloxacin, obstetric and gynecological doctors prescribed more norfloxacin, and urologists prescribed more ciprofloxacin and levofloxacin. There was no obvious prescription prevalence of fluoroquinolones for internists. Compared to those treated with TMP-SMX, patients treated with fluoroquinolones had higher burden of comorbidities, higher frequency of health care facilities utilization, and higher frequency of use of specific medications. However, there is little difference in baseline characteristics upon propensity score (PS) matching.

### Composite Treatment Outcome

Composite treatment outcome refers to outpatients experiencing treatment failure due to UTI-related hospitalization or UTI-related ED. Of 73,675 patients with UTI, 1420 (1.9%) patients were identified as having experienced composite treatment failure within 30 days of the index date. UTI-related hospitalization makes up of 65.3% (976) of the patients while UTI-related ED makes up of the reminder 35.7% (518) of the patients (Table 1). Composite treatment failure is experienced by 922 (1.7 %) of 54796 patients treated with TMP-SMX and 525 (2.8 %) of 18879 patients treated with a fluoroquinolone. However, there is difference in the crude incidence of treatment failure in different fluoroquinolone subgroup: ciprofloxacin 179/4184 (4.3%), levofloxacin 165/3142 (5.3%), ofloxacin 85/5984 (1.4%), and norfloxacin 69/5569 (1.2%). The odds ratios (ORs) for treatment failure based on multivariate logistic regression and propensity-score (PS) matching are presented in Table 2. Compared with TMP-SMX, norfloxacin had lowest treatment failure rate in crude estimate (OR, 0.73; 95% CI, 0.57–0.94), individual covariate adjustment (OR, 0.70; 95% CI, 0.54–0.90), and propensity score matched analysis (OR, 0.73; 95% CI, 0.54–1.00). Ofloxacin treatment when compared with TMP-SMX, was associated with significant lower risk for treatment failure in covariate-adjusted analysis (OR, 0.79; 95% CI, 0.62–0.99), but not in crude (OR, 0.84; 95% CI, 0.67–1.05) and PS-matched analysis (OR, 0.79; 95% CI, 0.59–1.05). Ciprofloxacin or levofloxacin treated patients, although shows significant higher unadjusted risk for treatment failure, does not show significantly higher treatment failure rate after covariate adjustment or PS matching. Both ofloxacin and norfloxacin show a significant lower rate for treatment failure as compared with ciprofloxacin treatment in crude, covariate-adjusted and PS-matched analysis. However, there is no significant difference in treatment failure rate of levofloxacin compared with ciprofloxacin, before and after the adjustment.

**TABLE 2:**
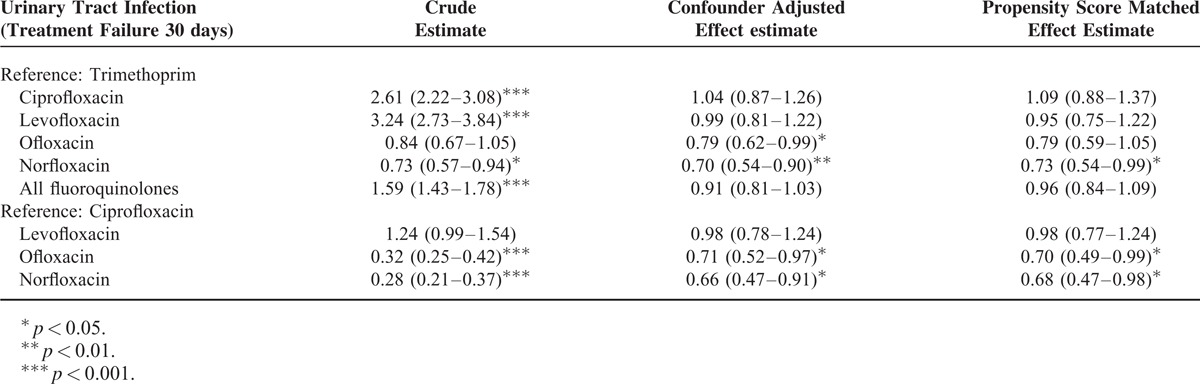
Comparison of Composite Treatment Failure Rates Between Different Antibiotic Regimens

### Sensitivity Analysis

To verify the robustness of the primary results and investigate the various influence of different definitions of treatment failure, we repeated the primary analyses on three different outcome definitions: UTI related ED visit within 30 days; UTI related hospitalization within 30 days; and composite UTI related ED visit/hospitalization within 42 days.

### Treatment Failure Defined as Hospitalization/ER Within 30 Days

Using this stricter definition of treatment failure defined as UTI related hospitalization within 30 days, Table 3 shows a result similar to the primary analysis. Specifically, after PS-matching, patients receiving norfloxacin (OR, 0.55; 95% CI, 0.67–1.05) or ofloxacin (OR, 0.68; 95% CI, 0.49–0.93) were less likely to experience an UTI-related hospitalization than patients receiving TMP-SMX. Patients receiving norfloxacin (PS-matched OR, 0.69; 95% CI, 0.45–1.06) and ofloxacin (PS-matched OR, 0.75; 95% CI, 0.51–1.05) are also less likely to experience an UTI-related hospitalization than patients receiving ciprofloxacin. However, the result is not significant after PS matching.

**TABLE 3:**
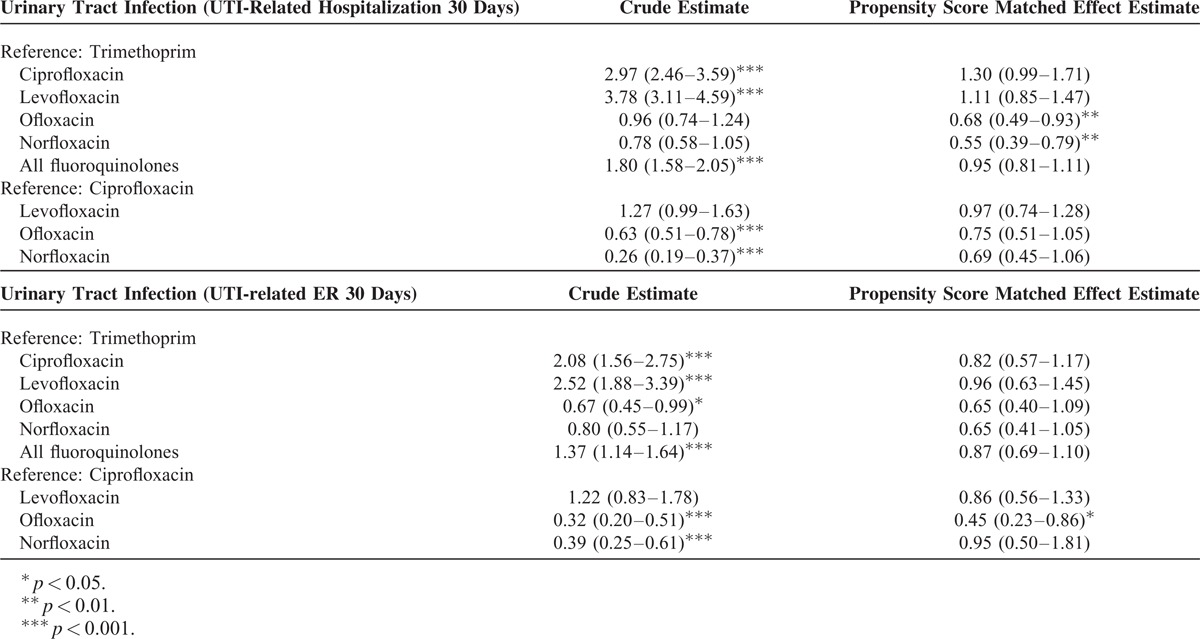
Sensitivity Analysis Using UTI-Related Hospitalization/ER as the Treatment Failure Outcome

For UTI-related ED visit within 30 days, results are also comparable to the primary analysis. Patients receiving norfloxacin (PS-matched OR, 0.65; 95% CI, 0.41–1.05) and ofloxacin (PS-matched OR, 0.65; 95% CI, 0.41–1.05) are less likely to have a UTI-related ED visit than patients receiving TMP-SMX, but the results are not significant. Compared with ciprofloxacin, only ofloxacin user has significant reduce risk in UTI-related ED visit before (OR, 0.32; 95% CI, 0.20–0.51) and after PS matching (OR, 0.45; 95% CI, 0.23–0.86).

### Treatment Failure Defined as Composite Treatment Failure within 42 Days

Extending the follow-up period to 42 days, results are still similar to the primary analysis of 30 days follow up (Table 4). Both norfloxacin and ofloxacin were associated with significant lower risk for treatment failure compared with TMP-SMX/ciprofloxacin before and after matching.

**TABLE 4:**
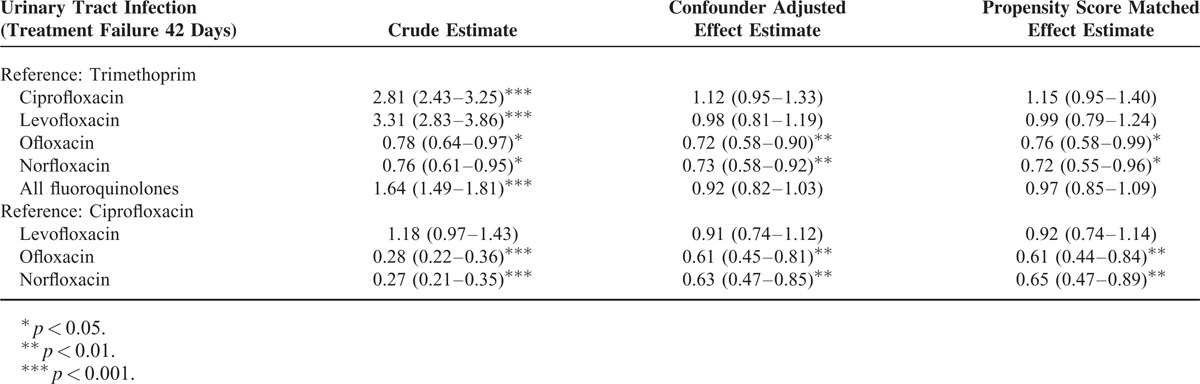
Sensitivity Analysis Using Different Duration of Follow-Up

### Subgroup Analysis

Table 5 shows the results of age, gender, indwelling catheters, bedridden status, and spinal cord injury subgroup analysis. For UTI patients greater than 60 years old, ofloxacin is the only antibiotic that can reduce the risk of treatment failure when compared to TMP-SMX (PS adjusted OR, 0.65; 95% CI, 0.44–0.95). When compared to TMP-SMX, norfloxacin is more effective on female patients (PS adjusted OR, 0.65; 95% CI, 0.48–0.88), patients without (W/O) indwelling catheters (PS adjusted OR, 0.73; 95% CI, 0.55–0.96), W/O bedridden status, and W/O spinal cord injury (PS adjusted OR, 0.72; 95% CI, 0.56–0.93). Compared with ciprofloxacin, both ofloxacin and norfloxacin can significantly reduce the risk of treatment failure in the female, W/O bedridden status and W/O spinal cord injury subgroups. When compared with ciprofloxacin, levofloxacin is the only drug that can significantly reduce the treatment failure in male patients (PS adjusted OR, 0.75; 95% CI, 0.61–0.94).

**TABLE 5:**
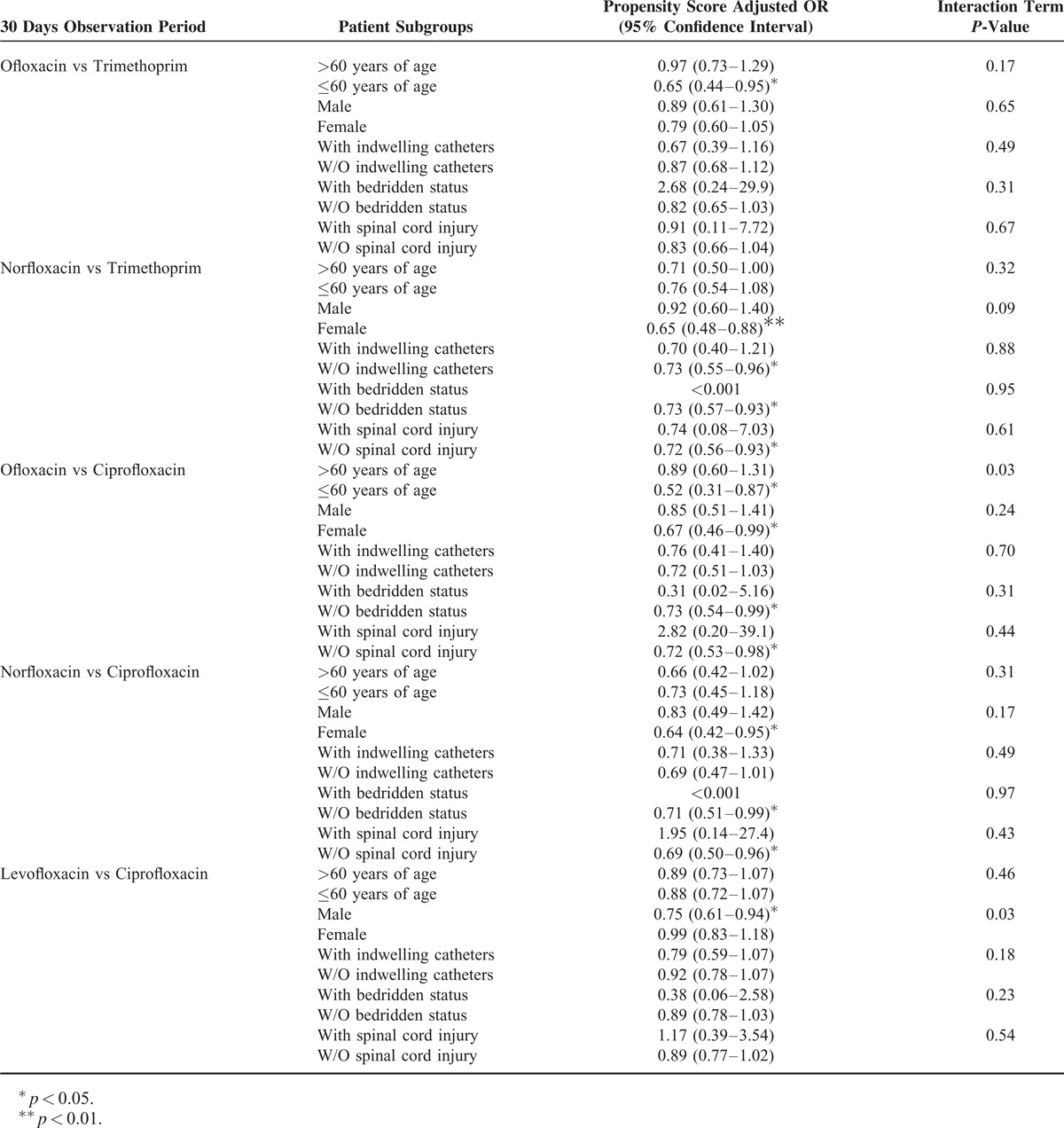
Comparative Risk for Treatment Failure in Different Susceptible Patient Subgroups

## DISCUSSION

In this claims analysis involving 73,675 patients with newly diagnosed UTI in an outpatient setting, the comorbidity patterns for patients receiving different fluoroquinolone subgroups are very different. After comprehensively adjusting for confounders by propensity score matching, those initially treated with norfloxacin or ofloxacin had lower rates of composite treatment failure than outpatients initially treated with TMP-SMX or ciprofloxacin. Surprisingly, levofloxacin, the most advanced fluoroquinolone, showed no significant difference in treatment failure rate when compared to TMP-SMX or ciprofloxacin. Similar results were obtained in sensitivity analysis using outcome definitions of UTI related ED visit within 30 days, UTI related hospitalization within 30 days, and composite UTI outcome within 42 days. Hence, confirming the robustness of the analysis. In general, subgroup analysis suggested that both norfloxacin and ofloxacin were more effective in female patients without complications (W/O indwelling catheters, W/O bedridden status, and W/O spinal cord injury), when compared with either TMP-SMX or ciprofloxacin.

There are limited real world clinical data on the comparative effectiveness of different antibiotic regimens for the treatment of UTIs. As far as we are aware of, there is only one comparative effectiveness study comparing TMP-SMX to a pool of two fluoroquinolone subgroups (ciprofloxacin and norfloxacin).^23^ Unfortunately, the Carrie group carried out their study 10 years ago. The constantly changing resistance rates of TMP-SMX and fluoroquinolone subgroups made this data of limited clinical relevance.^24–27^ In addition, the Carrie group defined refill of antibiotics and a change and/or addition to the initial antibiotics as criteria in treatment failure. We think it is difficult to establish the link between treatment failure and refill or change of antibiotics in an administrative database. This is because the indication for second antibiotics course is relatively hard to define, and the treatment duration of antibiotics is influenced by the physician perception and adherence to the recommended treatment guideline.

TMP-SMX has been recommended as the first line empirical antibiotic in IDSA consensus UTI treatment guidelines.^6,7^ However, the resistance rate of several uropathogens to TMP-SMX have exceeded 20% in Taiwan, Asia and many western countries.^24,27–29^ Fluoroquinolones have been proposed to be the new preferred empirical treatment for UTIs and their prescription number have steadily increased in Taiwan and the US.^24,30^ Various data have suggested the superiority of fluoroquinolone over TMP-SMX. Meta-analysis, clinical trials, in vitro resistance data and a comparative effectiveness research showed that UTI patients prescribed with fluoroquinolone had better outcome than patient prescribed with TMP-SMX.^23,24,28,31–34^ Hence, it is not surprising that patients treated with norfloxacin or ofloxacin had lower rates of composite treatment failure than patients initially treated with TMP-SMX. However, we are the first group to report that ciprofloxacin and levofloxacin have about 3-fold increase in unadjusted treatment failure rate when compared to TMP-SMX. We attributed the high unadjusted treatment failure rate of ciprofloxacin and levofloxacin to their prescription pattern to patients with higher number of comorbidities. The crude estimates dropped almost 3-fold either by adjusting for individual confounder or by propensity score matching. After adjustment, the treatment failure rates showed no significant difference when compared to TMP-SMX. Taken with the strong data showing the superiority of fluoroquinolone over TMP-SMX, our data is best interpreted, as UTI patients with multiple comorbidities are likely to have high treatment failure rate regardless of the antibiotic of choice. Hence, there will be no difference in treatment failure rate for very sick patient prescribed with either levofloxacin or TMP-SMX.

To the best of our knowledge, the in vitro resistant data of uropathogens to different fluoroquinolone subgroups has not been reported in Taiwan. Hence, based on ethnic and regional similarity to South Korea, we drew inference from the only available in vitro resistant comparison of different fluoroquinolone subgroups.^29^ This Korean study showed that most fluoroquinolone subgroups have similar drug susceptibility towards *Escherichia coli* and *Klebsiella pneumoniae*. However, norfloxacin has higher drug susceptibility than ciprofloxacin for *Proteus mirabilis* (100% vs 80.9%), and *Acinetobacter baumannii* (83.3% vs 48.6%). According to a recent antimicrobial resistance study, the respective incident rate of the top 5 causative uropathogens in both Taiwan and South Korea are: *E coli* [47.2% (Taiwan) and 63.8% (South Korea)], *K pneumoniae* (14.3% and 18.8%), *Pseudomonas aeruginosa* (8.8% and 3.8%), *P mirabilis* (8.0% and 1.3%), and *A baumannii* (4.8% and 0%).^35^ Using the above publications at face value, we hypothesized that our data showing the lower treatment failure rate of norfloxacin over ciprofloxacin might be due to higher drug susceptibility of *P mirabilis* and *A baumannii*.

The better patient outcome of norfloxacin over ciprofloxacin can also be explained by its usage indication. Fluoroquinolones with broad indication such as Ciprofloxacin are prescribed more often than Norfloxacin, which has specific indication for UTI. ^14^ In Taiwan, the total prescription number of ciprofloxacin, exceeds norfloxacin by 3-fold after year 2006 (Appendix 2). Increased usage of antibiotics has been found to correlate directly with the antibiotic resistant rate.^30^ Hence, we hypothesize that uropathogens are more resistant to commonly used fluoroquinolone such as Ciprofloxacin than Norfloxacin, which has specific indication.

The lower composite treatment failure rate of ofloxacin over ciprofloxacin can be also explained by in vitro resistant data and prescription pattern. According to the Korean report, ofloxacin has higher drug susceptibility than ciprofloxacin for *E coli* (50.2% vs 47.5%) and *A baumannii* (80.0% vs 48.6%).^29^ Hence, this might contribute to the lower treatment failure rate of ofloxacin as compared to ciprofloxacin. In addition, the increasing higher total prescription of ciprofloxacin over ofloxacin, might also cause the uropathogens to develop higher resistance for ciprofloxacin (Appendix 2).

Our study should be interpreted in the context of the limitations inherent in its design. A major limitation inherent in our study design is that we did not have information on patient behavior. Unrecorded events on the administrative database such as, patients missing the medications can lead to treatment failure. However, we have strict inclusion and exclusion criteria to minimize the human causes in treatment failure. First, we excluded subjects with signs of abnormal medication prescription, by eliminating patients with a prescription for an UTI-related antibiotic for more than 10 days. Second, to rule out the role of physician perception in influencing our results, we only analyzed UTI treatment failure outcome that can be clearly defined. Finally, to capture severity information, we collected the procedure codes as a proxy for UTI severity. These proxy indicators of severity correlate with comorbidity to a large extent. Hence, alleviating the concerns on the confounding by severity.

Since this is an observational study, our results are subjected to confounding. In order to make a fair comparison between patients with 47 different comorbidities, the propensity score matching technique is used.^36^ Propensity score matching is a well-documented method that addresses the sparse data problem when matching for individual covariates. In addition, propensity score can minimize the confounding bias by balancing the observable pretreatment characteristics between patients receiving two different treatments.

However, we cannot exclude the possibility that there are uncontrolled variables, even if we tried to be as comprehensive as possible. Our database lacks many personal variables that can lead to confounding. For instance, participants with unusual sexual behavior such as prostitutes carried a higher risk for UTI.^37^

To conclude, this is the first population-based comparative effectiveness research on UTI patients receiving different fluoroquinolone subgroups and TMP-SMX. Given the high TMP-SMX resistance rate as discussed earlier, our data suggest that patients prescribe with ofloxacin and norfloxacin might have better outcome when compared with TMP-SMX. However, results of this study cannot be readily generalized to other countries, as there are regional differences in antibiotic resistance patterns. More population-based comparative effectiveness studies are encouraged and should be considered as an integral piece of evidence in the local UTI treatment guideline.

## Appendix 1: Propensity Score Model, Using Levofloxacin as the Demonstration

[Table T1A]

### Appendix 2: Total Prescription Number of Different Fluoroquinolone

[Table T2A]
