# Comparative Effectiveness of Different Oral Antibiotics Regimens for Treatment of Urinary Tract Infection in Outpatients: An Analysis of National Representative Claims Database

**DOI:** 10.1097/01.md.0000464202.25179.0b

**Published:** 2015-01-16

An uncorrected version of “Comparative Effectiveness of Different Oral Antibiotics Regimens for Treatment of Urinary Tract Infection in Outpatients: An Analysis of National Representative Claims Database”^1^ was originally posted in Volume 93, Issue 28 of *Medicine*. The article has since been corrected and reposted in the issue.
